# Analysis of radiographic factors affecting the significant differences in knee alignment between hip-to-talus and hip-to-calcaneus radiographs after opening-wedge high tibial osteotomy

**DOI:** 10.1186/s43019-023-00203-4

**Published:** 2023-12-07

**Authors:** Hyung Jun Park, Joon Hyeok Boo, Dong Hun Suh, Jae Gyoon Kim

**Affiliations:** Department of Orthopedic Surgery, Korea University College of Medicine, Ansan Hospital, 123, Jeokgeum-Ro, Danwon-Gu, Ansan-Si, 425-707 Gyeongki-Do Korea

**Keywords:** Knee, Opening-wedge high tibial osteotomy, Hip-to-talus radiograph, Hip-to-calcaneus radiograph, Ankle joint line obliquity

## Abstract

**Background:**

Optimal alignment after opening-wedge high tibial osteotomy (OWHTO) is crucial for obtaining good clinical results. A hip-to-calcaneus radiograph (HCR) appears to reflect the true mechanical axis. However, no study has been reported using the HCR in patients who underwent OWHTO. We aimed to analyze the radiographic factors affecting the significant difference in the weight-bearing line (WBL) ratio between two radiographs after opening-wedge high tibial osteotomy (OWHTO).

**Methods:**

This retrospective study included 51 patients who underwent both hip-to-talus radiographs (HTR) and HCR after OWHTO. The patients were divided into two groups; a consistent group (WBL ratio difference between postoperative HTR and HCR < 5%; *N* = 35) and an inconsistent group (> 5%; *N* = 16). Radiographic variables for lower extremity alignment, knee and ankle joints, and clinical scores were evaluated. The receiver operating characteristic curve was used to determine the threshold of radiographic variables that induced inconsistencies between the two radiographs.

**Results:**

The mean postoperative WBL ratio in the HCR of the inconsistent group was significantly higher than that of the consistent group (57.7 ± 13.2% and 49.1 ± 11.6%, respectively) (*P* = 0.02). The preoperative and postoperative ankle joint line obliquity (AJLO) and preoperative lateral distal tibia ground surface angle (LDTGA) were significantly different between the two groups (*P* < 0.05). The preoperative AJLO (odds ratio 0.784, confidence interval 0.655–0.939, *P* = 0.008) significantly affected WBL ratio inconsistency. The cutoff value of the preoperative AJLO was 3.16°. However, clinical scores did not differ significantly between the two groups.

**Conclusion:**

The pre-and postoperative AJLO and the preoperative LDTGA were significantly different between the two groups. Among these variables, only preoperative AJLO negatively affected the inconsistency in WBL ratios between the two radiographs (HTT and HTC). Therefore, it should be considered to prevent postoperative overcorrection of the true mechanical axis after OWHTO, even though we corrected it properly.

*Level of evidence* Level IV.

**Supplementary Information:**

The online version contains supplementary material available at 10.1186/s43019-023-00203-4.

## Background

Optimal alignment after opening-wedge high tibial osteotomy (OWHTO) is crucial for obtaining good clinical results [1]. Previous studies have suggested that the optimal weight-bearing line (WBL) runs through 62.5–70% of the medial margin of the proximal tibia plateau after OWHTO [2, 3]. Precise and proper preoperative planning is necessary to obtain accurate alignment after OWHTO. Most surgeons use the conventional hip-to-talus radiograph (HTR) to plan properly, which shows the hip, knee, and ankle joints for preoperative planning. The reliability and availability of this method have been reported to be good [4, 5].

The concept of a “true mechanical axis” has been suggested [6]. The true mechanical axis of the lower extremity is the line from the center of the femoral head to the lowest point of the calcaneus and not to the tibial plafond. This mechanical axis is also known as the “ground mechanical axis” [7–9]. Some authors have suggested that the true mechanical axis may be a better measure than the conventional mechanical axis for elucidating lower extremity alignment [9]. The alignment of the hindfoot should be determined to evaluate the true mechanical axis because the loading axis passes through the lowest point of the calcaneus to the ground [7, 8]. However, we were unable to find the true mechanical axis using the conventional lower extremity radiograph (HTR), as we could not find the hindfoot alignment in this radiograph. A wire technique was proposed to determine the true mechanical axis on conventional lower extremity radiographs [7, 8]. However, this technique fixed a flexible wire to a point of soft tissue on the heel rather than directly on the calcaneus, [8] which could cause an error. Recently, Haraguchi et al. suggested a novel hip-to-calcaneus radiograph (HCR), which was able to show the hip, knee, and hindfoot together [6]. This radiography technique appears to be a simple and highly accurate method for evaluating the true mechanical axis [10].

Alignment of the knee joint is closely related to that of the ankle joint and hindfoot [11–13]. The varus malalignment of the knee joint leads to valgus compensation at the ankle and subtalar joints [11–13]. The compensation is higher in the subtalar joint than that in the ankle joint [12]. Correcting the varus malalignment of the knee joint causes the ankle joint to move into a varus position, and the subtalar joint is compensated to move into a varus position [12, 14, 15]. However, if the subtalar joint is stiff, the compensation does not occur properly, which causes pain around the ankle joint [16, 17]. In addition, a stiff subtalar joint leads to a deviation of the true mechanical axis from the conventional mechanical axis. One study reported that although varus malalignment of the knee was restored after total knee arthroplasty (TKA), the true mechanical axis would pass lateral to the center of the knee joint if preoperative hindfoot valgus alignment persisted after surgery [8]. In a previous study, we presented the possibility of “true mechanical axis” deviation compared with the conventional mechanical axis. The “true mechanical axis” and the conventional mechanical axis were measured using HCR and HTR, respectively. Our results showed that the true mechanical axis differed significantly from the conventional mechanical axis in patients with larger genu varum deformity [10].

Based on this knowledge, we hypothesized that the true mechanical axis would be different from the conventional mechanical axis, even though we corrected it properly following the preoperative planning using the conventional mechanical axis in HTR after OWHTO. Therefore, this study aimed to compare the true mechanical axis in the HCR to the conventional mechanical axis in the HTR after OWHTO and to analyze the radiographic factors affecting the difference in this mechanical axis deviation measured by the WBL ratio of the knee joint between the HTR and HCR after OWHTO.

## Methods

### Study design and population

**Fig. 1:**
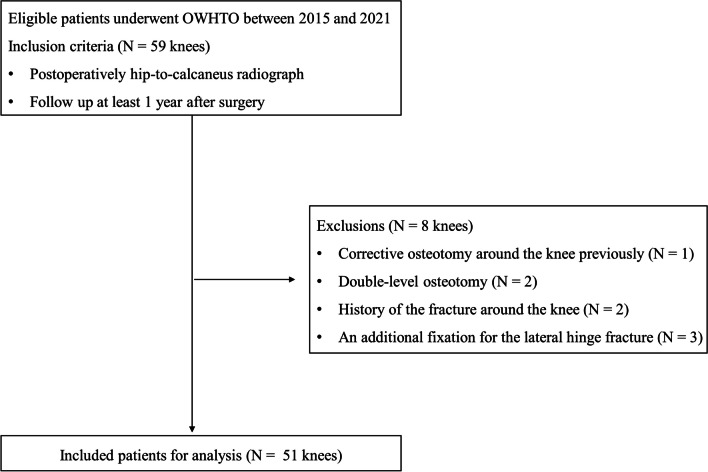
Selection of the patients for the study. *OWHTO* opening-wedge high tibial osteotomy, *N* number

Among the patients who underwent OWHTO from 2015 to 2021, those who took both HTR and HCR postoperatively and were followed up for at least 2 years after surgery were included. Among the eligible patients, eight patients were excluded as per the following exclusion criteria: [1] patients who had previously undergone corrective osteotomy around the knee [number (*N*) = 1), those who had undergone double-level osteotomy (*N* = 2), those who had a history of the fracture around the knee (*N* = 2), and those who needed an additional fixation for the lateral hinge fracture (*N* = 3). Finally, 51 knees were included in this study (Fig. 1). This study was approved by our institutional review board before analyzing the data retrospectively.

### Surgical technique

All OWHTO procedures were performed using the same surgical technique by a single orthopedic surgeon. The target point for alignment correction was 62.5% of the medial edge of the proximal tibia plateau in the HTR. The degree of correction was measured using Miniaci’s method with the HTR [18, 19]. All patients underwent arthroscopic procedures to determine intra-articular pathologies before osteotomy. A longitudinal incision was made on the anteromedial side of the proximal tibia. After the pes anserinus was retracted, the superficial medial collateral ligament was detached from the distal insertion site of the proximal tibia [20]. Osteotomy was performed using a biplanar technique. Two k-wires were inserted approximately 4 cm below the knee joint line toward the tip of the fibular head. Distal osteotomy was performed along the two inserted K-wires. Proximal osteotomy was performed at 110° from the distal osteotomy line [21]. The valgus force was applied to the osteotomy site using a three-chisel technique. After confirming that the lower extremity alignment was corrected to the desired angle, the osteotomy site was fixed using a locking plate (OhtoFix; Ohtomedical, Goyang, Republic of Korea / TomoFix; De Puy Synthes, Raynham, MA) [22].

### Radiographic and clinical evaluations

**Fig. 2:**
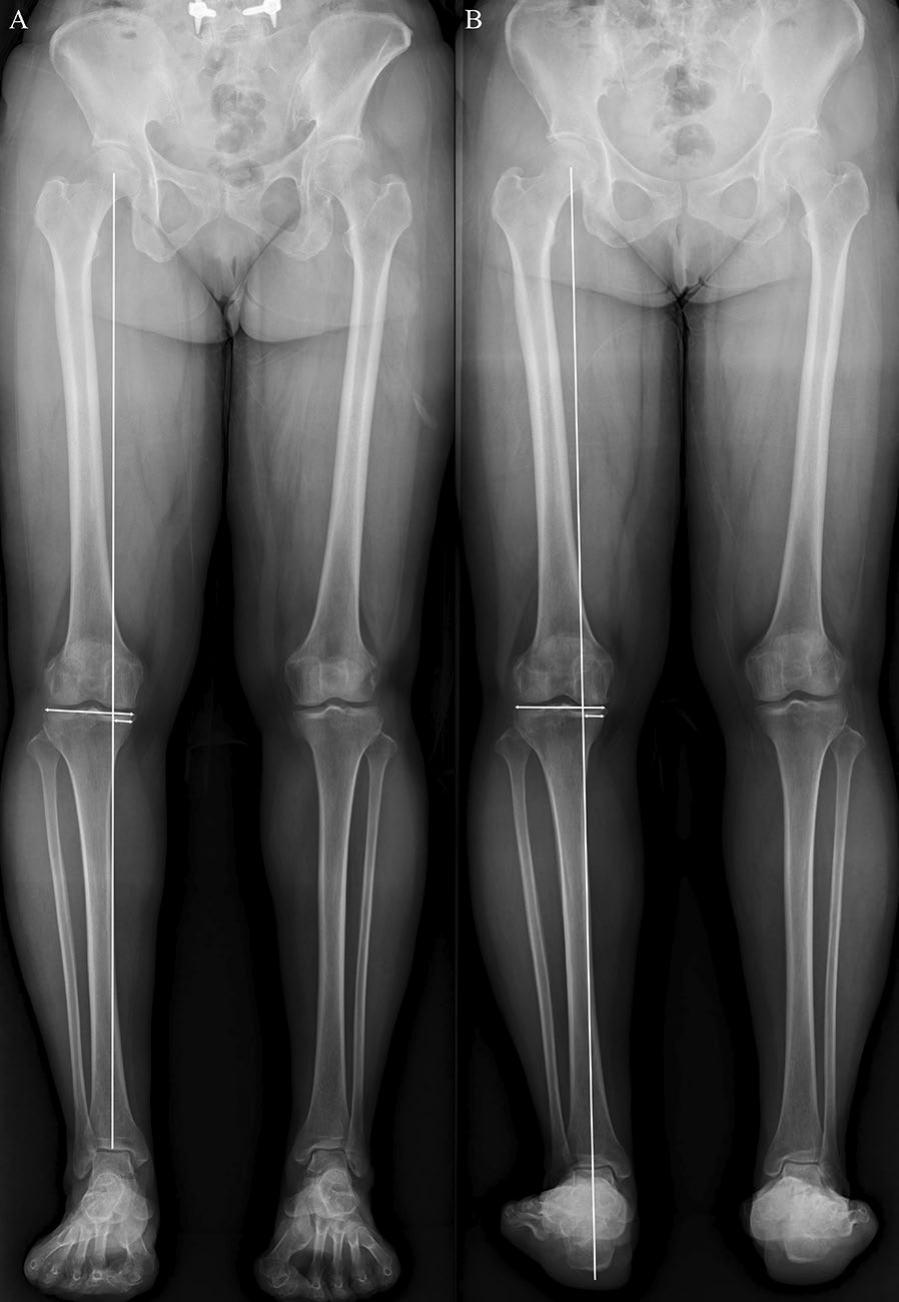
Weight-bearing line (WBL) ratio on the two lower extremity radiographs. The WBL line was drawn from the center of the femoral head to the center of the superior surface of the talus (**A** hip-to-talus radiograph) or the lowest point of the calcaneus (**B** hip-to-calcaneus radiograph). The WBL ratio was determined as the percentage by dividing the length from the medial portion of the proximal tibia plateau to the point where WBL crossed the proximal tibia plateau by the total length of the proximal tibia plateau

**Fig. 3:**
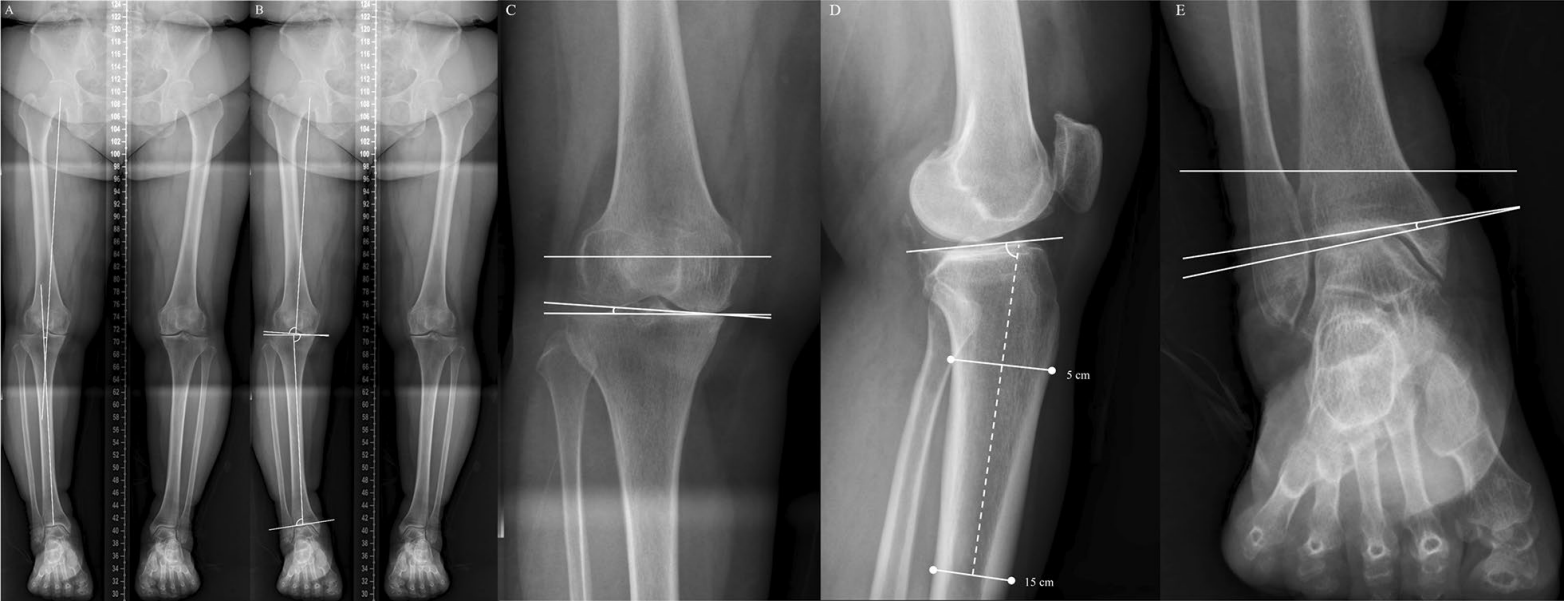
Radiographic variables for the alignment of the lower extremity, the knee and ankle. **A** The hip-knee-ankle angle was determined as the angle between the mechanical axis of the femur and tibia. **B** Mechanical lateral distal femoral angle was determined as the angle between the mechanical axis of the femur and the line connecting distal femoral condyles. Medial proximal tibia angle was determined as the angle between the mechanical axis of the tibia and the tangent line of the proximal tibial plateau. Mechanical lateral distal tibial angle was determined as the angle between the mechanical axis of the tibia and the tangent line of the superior surface of the talus. **C** Joint line convergence angle was determined as the angle between the tangent lines of the distal femoral condyles and the proximal tibia plateau. Knee joint line obliquity was defined as the angle between the tangent line of the proximal tibial plateau and the ground surface line. **D** Posterior tibial slope was determined as the angle between the proximal tibial plateau and the line connecting the midpoint of 5 cm and 15 cm distal from to knee joint line. **E** Talar tilt angle was determined as the angle between the tangent lines of the distal surface of the tibia and the superior surface of the talus. Lateral distal tibial ground surface angle was defined as the angle between tangential line of the distal surface of the tibia and the ground surface line. Ankle joint line obliquity was determined as the angle between the tangent line of the superior surface of the talus and the ground surface line

All patients underwent standing knee anteroposterior, standing knee lateral, and lower extremity radiographs preoperatively and postoperatively. The lower extremity radiographs were obtained using two techniques: [1] HTR and [2] HCR [6, 10]. The HTRs of the lower extremity were taken anterior to posterior direction as the conventional method [4]. In contrast, the HCRs of the lower extremity were taken posterior to anterior direction, and the lowest point of the calcaneus, which was found to be the contact point of the calcaneus with the ground surface on the radiographs, was shown [6, 10]. After confirming that the lower extremities were not rotated, the radiographic variables were evaluated [6]. WBL was drawn in both HTR and HCR from the center of the femoral head to the center of the superior surface of the talus or the lowest point of the calcaneus, respectively [10, 23]. The WBL ratio was determined as the percentage by dividing the length from the medial portion of the proximal tibia plateau to the point where WBL crossed the proximal tibia plateau by the total length of the proximal tibia plateau [10, 24] (Fig. 2). The hip-knee-ankle angle was determined as the angle between the mechanical axis of the femur and tibia using HTR [25]. Mechanical lateral distal femoral angle (mLDFA) was determined as the angle between the mechanical axis of the femur and the line connecting the distal femoral condyles using HTR [26, 27]. Medial proximal tibia angle (MPTA) was determined as the angle between the mechanical axis of the tibia and the tangent line of the proximal tibial plateau using the HTR [26, 27]. Mechanical lateral distal tibial angle (mLDTA) was determined as the angle between the mechanical axis of the tibia and the tangent line of the superior surface of the talus using the HTR [26, 27] (Fig. 3). We also measured radiographic variables around the knee and ankle joints using standing knee anteroposterior, standing knee lateral, and HTR. The joint line convergence angle (JLCA) was determined as the angle between the tangent lines of the distal femoral condyles and the proximal tibia plateau [23]. Knee joint line obliquity (KLCA) was defined as the angle between the tangent line of the proximal tibial plateau and the ground surface line [28]. Posterior tibial slope (PTS) was determined as the angle between the proximal tibial plateau and the line connecting the midpoint of 5 cm and 15 cm distal to the knee joint line on the standing lateral radiograph [29]. For the radiographic variables around the ankle joint, talar tilt angle (TTA) was determined as the angle between the tangent lines of the distal surface of the tibia and the superior surface of the talus [17]. Lateral distal tibial ground surface angle (LDTGA) was defined as the angle between the tangential line of the distal surface of the tibia and ground surface line [17]. Ankle joint line obliquity (AJLO) was determined as the angle between the tangent line of the superior surface of the talus and the ground surface line [17, 30] (Fig. 3). These radiographic measurements were determined as positive values when the apex of the angle was medial. Differences between preoperative and postoperative radiographic measurements were also determined.

The Western Ontario and McMaster University Osteoarthritis Index (WOMAC), Knee Society Score (KSS), and Knee Injury and Osteoarthritis Outcome Score (KOOS) were used to evaluate clinical outcomes [31–33]. Patients were followed up at 6 weeks, 3 months, 6 months, 1 year, and every year postoperatively. The clinical scores recorded at the last follow-up were included in this study.

Patients were divided into two groups. If the difference in the WBL ratio between the HTR and HCR groups was < 5%, patients were classified into the consistent group, and if the difference was > 5%, they were classified into the inconsistent group following our previous study [10]. No significant differences were observed between the two groups in terms of demographic data (Table 1).

**Table 1:** Baseline characteristics of the included patients

	Consistent group (*N* = 35)	Inconsistent group (*N* = 17)	*P*-value
Age (years)*	54.5 ± 5.1	56.4 ± 5.2	0.226^†^
Sex			
Female; *N* (%)	25 (71.4)	12 (70.6)	0.597^‡^
Height (cm)*	159.1 ± 8.0	160.9 ± 5.9	0.417^†^
Weight (kg)*	70.4 ± 9.2	69.5 ± 10.0	0.771^†^
BMI (kg/m^2^)*	27.8 ± 3.3	26.9 ± 3.6	0.349^†^
Laterality			
Rt; *N* (%)	18 (51.4)	5 (29.4)	0.114^‡^
K/L, *N* (%)			
Grade 2	3 (8.6)	0 (0)	0.454^§^
Grade 3	27 (77.1)	14 (82.4)	
Grade 4	5 (14.3)	3 (17.6)	

*Values are presented as mean ± standard deviation

*N* number, *BMI* body mass index, *Rt* right, *K/L* Kellgren–Lawrence grade

Statistical analysis: ^†^independent sample t-test, ^‡^Chi-square test, ^§^Pearson Chi-square test

### Reliability and statistical analysis

Two orthopedic surgeons agreed and were trained in the measurement methods together; however, they were blinded to each other’s measurements and prior measurements. The reliability of the measurements was evaluated using intra- and interclass correlation coefficients. Independent t-tests, paired t-tests, and chi-square tests were performed to analyze patient characteristics and compare radiographic measurements. Correlations between radiographic variables and the inconsistency in WBL ratios measured by the two radiographs were statistically analyzed using univariate logistic regression analysis. Multivariate logistic regression analysis was also used to elucidate the preoperative radiographic factors for inconsistencies in WBL ratios. A receiver operating characteristic curve with the Youden index was used to determine the threshold of the preoperative radiographic variables that induced the inconsistencies in the WBL ratios between two radiographs. WOMAC, KSS, and KOOS scores were compared between the consistent and inconsistent groups. All data were analyzed using SPSS version 20 (SPSS Inc., Chicago, IL, USA) and power analysis was performed using G-power version 3.1.9.7 (Franz Faul, Germany). Statistical significance was set at *P*-value < 0.05.

## Results

The inter-observer correlation coefficient ranged from 0.83 to 0.99, and the intra-observer correlation coefficient ranged from 0.80 to 0.99. (Additional file 1: Appendix) The statistical power to compare the preoperative AJLO between the two groups was 0.93.

The mean preoperative WBL ratio of total cases in HTR and HCR was 18.2 ± 7.6% and 18.2 ± 10.0%, respectively. The mean postoperative WBL ratio of total cases in HTR and HCR was 50.4 ± 10.7% and 51.8 ± 12.6%, respectively. (*P* > 0.05) However, the mean postoperative WBL ratio in the HCR of the inconsistent group (57.7 ± 13.2%) was significantly greater (more lateral position of WBL) than that of the consistent group (49.1 ± 11.6%). There was a significant difference in the postoperative WBL ratio of the HCR between the two groups (*P* = 0.02). However, no significant differences were found in radiographic variables in lower extremity alignment and the knee joint (preoperative, postoperative, and difference) (*P* > 0.05). However, some radiographic variables about ankle joint were significantly different between the two groups. A significant difference was found in the preoperative AJLO (*P* = 0.004), preoperative LDTGA (*P* = 0.011), and postoperative AJLO (*P* = 0.042) (Table 2).

**Table 2:** Comparison in radiographic indexes between the consistent group and the inconsistent group

	Consistent group (*N* = 35)	Inconsistent group (*N* = 17)	*P*-value
Lower extremity alignment			
Preoperative			
HKA	7.2 ± 2.6	6.9 ± 2.3	0.726
mLDFA	89.2 ± 2.6	88.6 ± 1.7	0.429
MPTA	84.9 ± 2.1	84.8 ± 1.8	0.948
mLDTA	91.0 ± 3.0	89.8 ± 2.5	0.158
Postoperative			
HKA	0.18 ± 2.4	− 1.2 ± 2.8	0.082
mLDFA	88.9 ± 2.6	88.3 ± 1.6	0.365
MPTA	91.1 ± 2.8	91.8 ± 2.6	0.413
mLDTA	91.0 ± 3.1	88.8 ± 2.5	0.180
Difference*			
HKA	− 7.0 ± 3.0	− 7.7 ± 2.9	0.403
mLDFA	− 0.3 ± 1.0	− 0.3 ± 0.6	0.790
MPTA	6.3 ± 2.7	7.0 ± 2.5	0.365
mLDTA	− 0.5 ± 2.0	0.3 ± 2.0	0.214
Knee			
Preoperative			
JLCA	3.0 ± 2.3	3.0 ± 1.3	0.863
KJLO	− 0.4 ± 2.3	− 0.6 ± 2.1	0.780
PTS	10.7 ± 3.1	11.5 ± 2.9	0.361
Postoperative			
JLCA	2.4 ± 2.2	2.5 ± 1.5	0.883
KJLO	2.4 ± 3.0	2.2 ± 2.2	0.961
PTS	9.4 ± 3.3	9.6 ± 2.8	0.851
Difference *			
JLCO	− 0.6 ± 1.5	− 0.6 ± 1.2	0.965
KJLO	2.6 ± 2.3	2.8 ± 1.7	0.818
PTS	− 1.4 ± 2.9	− 1.9 ± 3.2	0.551
Ankle			
Preoperative			
TTA	**1.7 ± 1.9**	**0.2 ± 1.0**	**0.005**
AJLO	**7.9 ± 4.4**	**4.7 ± 3.7**	**0.011**
LDTGA	6.3 ± 3.8	4.4 ± 3.9	0.111
Postoperative			
TTA	1.0 ± 1.7	0.3 ± 1.2	0.147
AJLO	**3.0 ± 4.0**	**0.4 ± 3.0**	**0.022**
LDTGA	2.1 ± 3.9	0.3 ± 3.3	0.078
Difference*			
TTA	− **0.7 ± 1.1**	**0.1 ± 0.6**	**0.008**
AJLO	− 4.9 ± 2.8	− 4.2 ± 3.2	0.449
LDTGA	− 4.1 ± 2.5	− 4.2 ± 3.1	0.965

Values are presented as mean ± standard deviation, Statistically significant variables were expressed in bold

*Difference between postoperative and preoperative values

*N* number, *HKA* hip knee ankle axis, *MPTA* medial proximal tibial angle, *JLCA* joint line convergence angle, *KJLO* knee joint line obliquity, *PTS* posterior tibial slope, *TTA* talar tilt angle, *AJLO* ankle joint line obliquity, *LDTGA* lateral distal tibial ground surface angle

Statistical analysis: independent sample t-test

**Fig. 4:**
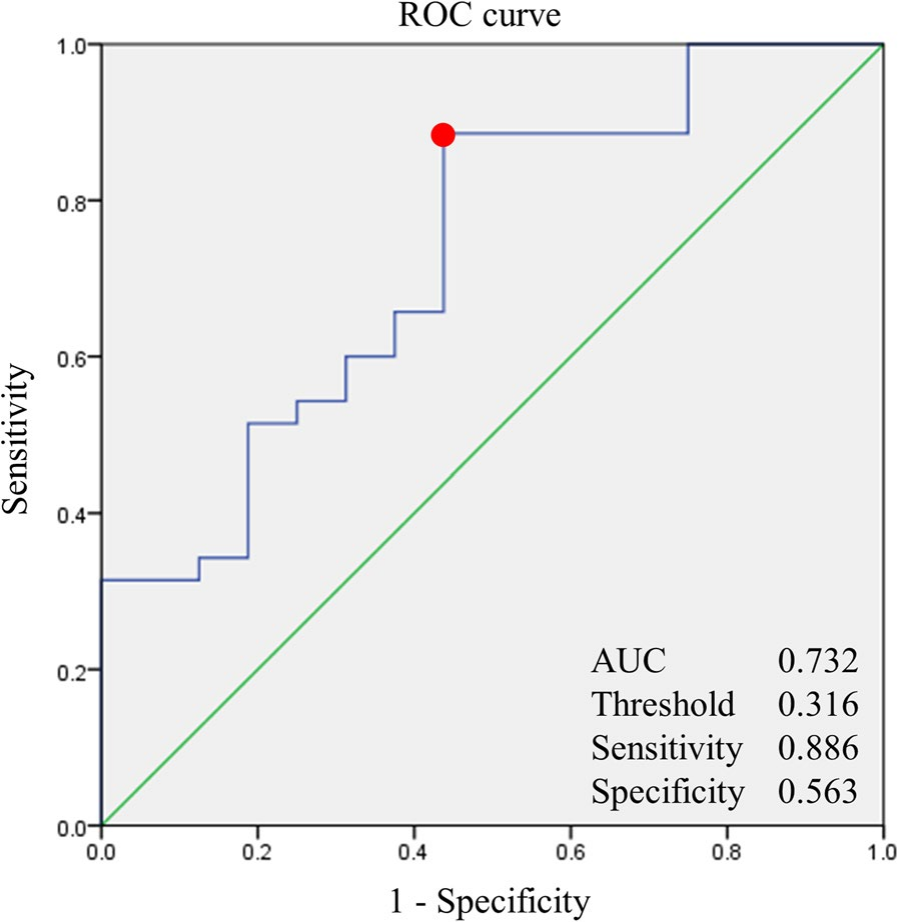
A receiver operating characteristic curve was used to determine the threshold of preoperative ankle joint line obliquity that induced the inconsistencies between the weight-bearing line ratios measured on the hip-to-talus radiograph and hip-to-calcaneus radiograph

Univariable logistic regression analysis showed preoperative AJLO [odds ratio (OR) 0.784; confidence interval (CI) 0.655–0.939, *P* = 0.008], preoperative LDTGA (OR 0.801; CI 0.667–0.962, *P* = 0.018) and postoperative AJLO (OR 0.839; CI 0.705–0.998, *P* = 0.048) were significant variables. Multivariate logistic regression analysis of the variables affecting the inconsistency in WBL ratios indicated that preoperative AJLO (OR 0.784; CI 0.655–0.939, *P* = 0.008) was the only significant variable (Table 3). The cut-off value of the preoperative AJLO that caused the inconsistency between the two radiographs was 3.16° (area under curve 0.732). The sensitivity and specificity were 0.886 and 0.563, respectively. (Fig. 4). The pre-and postoperative WOMAC, KSS, and KOOS scores were not significantly different between the consistent and inconsistent groups (*P* > 0.05) (Table 4).

**Table 3:** Risk factors for inconsistency between the WBL ratios measured on the two lower extremity radiographs (HTRs and HCRs)

	Univariate analysis		Multivariate analysis	
	OR	*P*-value	OR	*P*-value
Preoperative				
HKA	0.958 (0.758–1.211)	0.720		
MPTA	0.990 (0.738–1.329)	0.946		
mLDFA	0.898 (0.689–1.169)	0.423		
mLDTA	0.824 (0.675–1.005)	0.056		
JLCA	1.026 (0.772–1.364)	0.860		
KJLO	0.962 (0.738–1.254)	0.775		
PTS	1.099 (0.899–1.343)	0.355		
TTA	**0.406 (0.209–0.788)**	**0.008**	**0.406 (0.209–0.788)**	**0.008**
AJLO	**0.814 (0.689–0.963)**	**0.016**		0.143
LDTGA	0.882 (0.750–1.038)	0.130		
Postoperative				
HKA	0.816 (0.643–1.035)	0.094		
MPTA	1.100 (0.879–1.376)	0.406		
mLDFA	0.880 (0.668–1.158)	0.361		
mLDTA	0.870 (0.710–1.067)	0.181		
JLCA	1.023 (0.762–1.374)	0.880		
KJLO	0.994 (0.802–1.233)	0.960		
PTS	1.019 (0.843–1.231)	0.847		
TTA	0.731 (0.477–1.121)	0.151		
AJLO	**0.822 (0.690–0.980)**	**0.029**		0.160
LDTGA	0.849 (0.706–1.022)	0.084		
Difference*				
HKA	0.916 (0.747–1.122)	0.397		
MPTA	1.116 (0.883–1.411)	0.359		
mLDFA	0.911 (0.465–1.784)	0.785		
mLDTA	1.204 (0.898–1.615)	0.215		
JLCA	0.991 (0.656–1.495)	0.964		
KJLO	1.034 (0.781–1.370)	0.814		
PTS	0.939 (0.767–1.150)	0.544		
TTA	**3.682 (1.250–10.849)**	**0.018**		0.057
AJLO	1.084 (0.883–1.331)	0.442		
LDTGA	1.005 (0.808–1.249)	0.965		

*Difference between postoperative and preoperative values, Statistically significant variables were expressed in bold

*WBL* weight-bearing line, *HTR* hip-to-talus radiograph, *HCR* hip-to-calcaneus radiograph, *HKA* hip knee ankle axis, *MPTA* medial proximal tibial angle, *JLCA* joint line convergence angle, *KJLO* knee joint line obliquity, *PTS* posterior tibial slope, *TTA* talar tilt angle, *AJLO* ankle joint line obliquity, *LDTGA* lateral distal tibial ground surface angle

Statistical analysis: logistic regression analysis

**Table 4:** Comparison in clinical indexes between the consistent group and the inconsistent group

	Consistent group (*N* = 35)	Inconsistent group (*N* = 17)	*P*-value
Preoperative			
WOMAC	61.9 ± 14.2	52.3 ± 11.3	0.070
KSS_ knee	59.2 ± 16.3	70.5 ± 21.5	0.096
KSS_ function	58.8 ± 18.8	66.5 ± 16.7	0.267
HSS	64.7 ± 13.9	70.6 ± 16.5	0.780
KOOS_ pain	42.4 ± 16.9	50.9 ± 16.1	0.157
KOOS_ symptom	44.2 ± 17.3	51.7 ± 16.5	0.222
KOOS_ activity of daily living	41.3 ± 17.9	45.3 ± 15.2	0.509
KOOS_ sports	14.2 ± 20.9	22.7 ± 12.2	0.214
KOOS_ quality of life	18.8 ± 13.5	28.9 ± 16.4	0.057
Postoperative			
WOMAC	32.0 ± 17.9	30.4 ± 22.8	0.798
KSS_ knee	76.5 ± 15.9	77.5 ± 16.4	0.848
KSS_ function	73.2 ± 17.0	70.7 ± 10.3	0.599
HSS	76.0 ± 13.0	72.6 ± 12.9	0.406
KOOS_ pain	65.2 ± 20.2	71.5 ± 20.2	0.321
KOOS_ symptom	66.6 ± 18.8	67.7 ± 20.6	0.852
KOOS_ activity of daily living	68.0 ± 21.7	70.3 ± 23.7	0.747
KOOS_ sports	30.3 ± 24.3	41.3 ± 33.7	0.207
KOOS_ quality of life	44.9 ± 24.6	50.9 ± 30.6	0.473

Values are presented as mean ± standard deviation

*N* number, *WOMAC* Western Ontario and McMaster Universities Osteoarthritis Index, *KSS* Knee Society Score, *KOOS* Knee injury and Osteoarthritis Outcome Score

## Discussion

The principal finding of our study was that only the ankle joint variables were significantly different between the consistent and inconsistent groups. In addition, preoperative AJLO was a significant factor affecting the inconsistency in the WBL ratios between the two groups. The cut-off value for the preoperative AJLO that caused inconsistency in the WBL ratio between the two radiographs was 3.16°. Previous studies have evaluated the effect of a variety of knee surgeries on the ankle and subtalar joints [8, 23, 34–40]. However, there have been limited studies elucidating the effect of OWHTO on the ankle and subtalar joint [23, 34, 35, 37]. Some studies have evaluated the true mechanical axis deviation compared to the conventional mechanical axis using the HCR after TKA and unicompartmental knee arthroplasty (UKA) [8, 36]. However, to the best of our knowledge, no study has evaluated the radiographic factors affecting the difference in WBL ratio after OWHTO using the HCR compared to the HTR.

Hindfoot alignment should also be considered when evaluating the mechanical axis. The conventional mechanical axis, which runs from the femoral head to the center of the tibial plafond, was used to assess the lower extremity alignment. However, this mechanical axis does not consider the hindfoot alignment which transfers the load to the ground. Haraguchi et al. defined the true mechanical axis of the lower extremity as a line from the femoral head center to the contact point of the calcaneus with the ground, rather than to the center of the tibia plafond [6]. Guichet et al. described the importance of the hindfoot alignment in assessing the loading axis of the lower extremity. They reported a difference between the conventional mechanical axis and true mechanical axes, similar to the study of Haraguchi [7]. Furthermore, the true mechanical axis assessed using the HCR was found to be useful for evaluating knee and ankle joint kinematics during gait analysis. Kikuchi et al. reported the hip-to-calcaneal (HC) line reflected knee and ankle kinetics better than the hip-to-ankle (HA) line [41]. Matsumoto et al. compared HC line and HA lines using kinematically and mechanically aligned TKA. They suggested that the HC line passed through the center more than the HA line in patients undergoing kinematically aligned TKA [42].

**Fig. 5:**
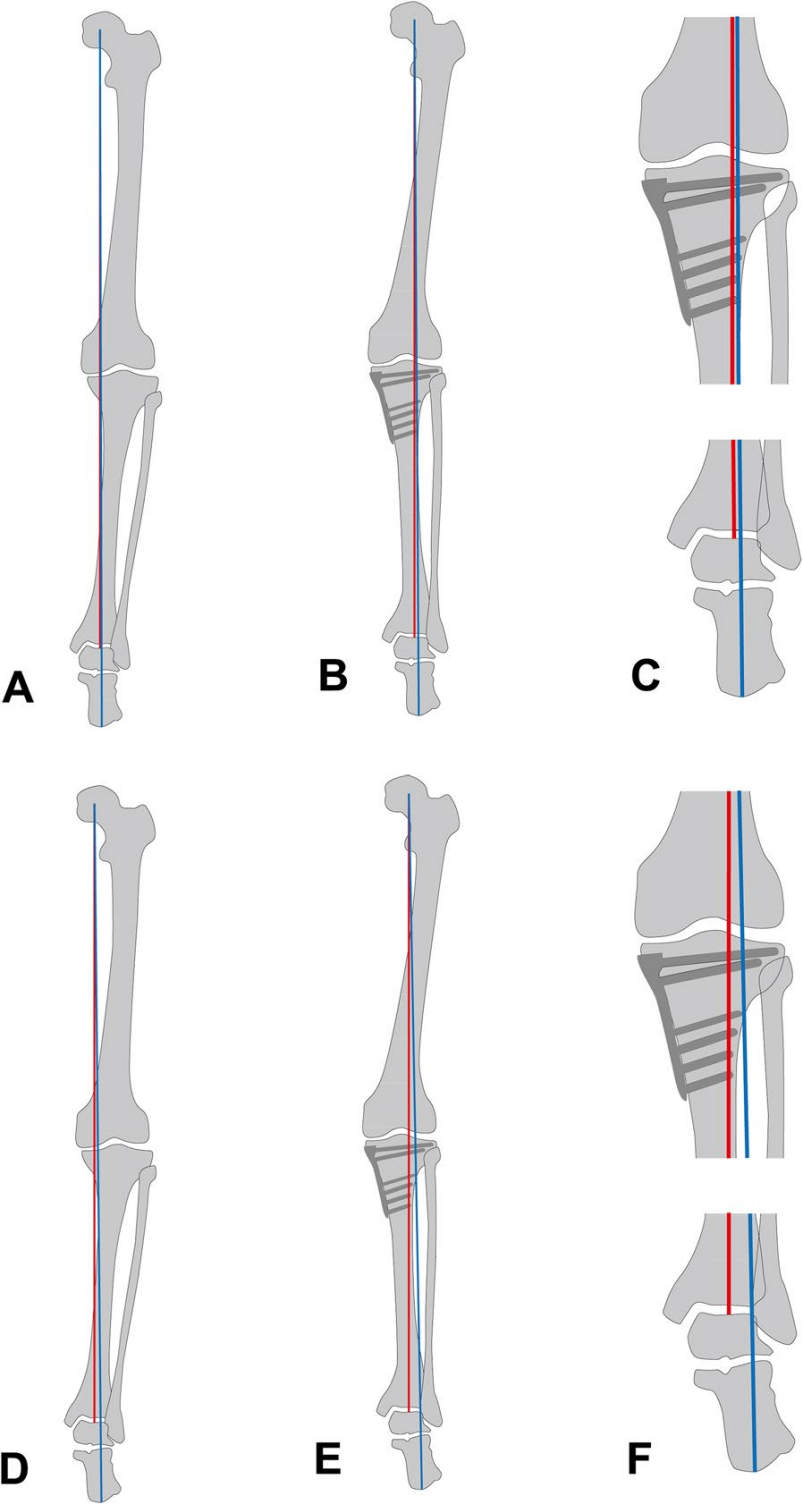
The illustrations show the conventional mechanical axis and true mechanical axis. **A** A preoperative left lower extremity with genu varum with the normal subtalar joint. **B** A postoperative left lower extremity after opening-wedge high tibial osteotomy (OWHTO), which does not show a difference between conventional and true mechanical axis. **C** A magnified illustration of the knee and ankle joints after OWHTO. **D** A preoperative left lower extremity with genu varum with the stiff subtalar joint. **E** A postoperative left lower extremity after OWHTO, which shows the difference between conventional and true mechanical axis. **F** A magnified illustration of the knee and ankle joints after OWHTO

Differences were found between the true and conventional mechanical axes after variable knee surgery. Kuroda et al. found that the true mechanical axis significantly showed more valgus alignment (approximately 1°) than the conventional mechanical axis after UKA for osteoarthritis with varus deformity. They also suggested that neutral alignment in the conventional mechanical axis corresponds to valgus alignment in the true mechanical axis, which might result in overcorrection, even though we corrected neutrally after UKA [36]. Mullaji et al. also demonstrated similar results in that the conventional mechanical axis showed less deviation from the center of the knee than the true mechanical axis, which exhibited lateral deviation after TKA [8]. They found that the hindfoot valgus alignment decreased after TKA, but approximately 87% of patients retained hindfoot valgus alignment, which caused lateral deviation of the true mechanical axis, in contrast to the conventional mechanical axis [8]. In our results, the WBL ratio of the total cases in the HCR was also greater (more lateral) than that in the HTR after OWHTO, consistent with previous studies, although the difference was not statistically significant [8, 36]. Based on these results, we determined that the true mechanical axis became lateral to the conventional axis following the correction of the varus malalignment of the knee. Although hindfoot alignment was not evaluated in our study, the remaining hindfoot valgus after OWHTO may have contributed to the high laterality of the WBL in HCR (Fig. 5). It is unclear why compensation did not occur at the subtalar joint; however, some authors have reported large preoperative hindfoot valgus alignment, [8] preoperative hindfoot varus alignment, [14] severe preoperative genu varum, [43] and severe ankle osteoarthritis with subtalar joint stiffness [35, 44].

**Fig. 6:**
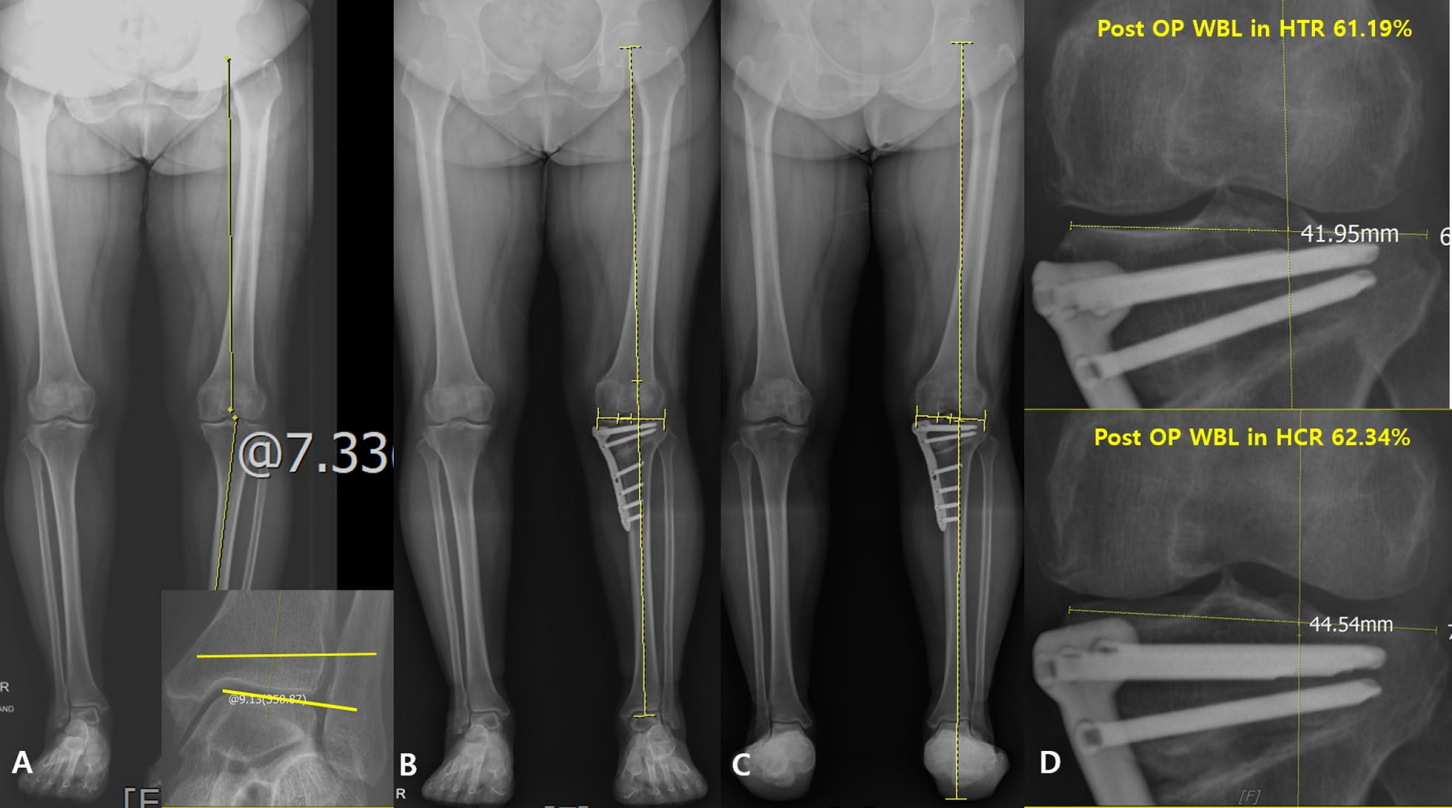
**A** 61-year-old male patient showed left knee osteoarthritis with hip-knee-ankle angle 7.3° genu varum deformity. The ankle joint line obliquity was 9.13°. **B** The conventional mechanical axis after opening-wedge high tibial osteotomy (OWHTO). **C** The true mechanical axis after OWHTO. **D** The postoperative weight-bearing line (WBL) ratio in the hip-to-talus radiograph after OWHTO was 61.1%, and that of the WBL ratio in the hip-to-calcaneus radiograph after OWHTO was 62.3%. There was no significant difference between the conventional mechanical axis and the true mechanical axis

**Fig. 7:**
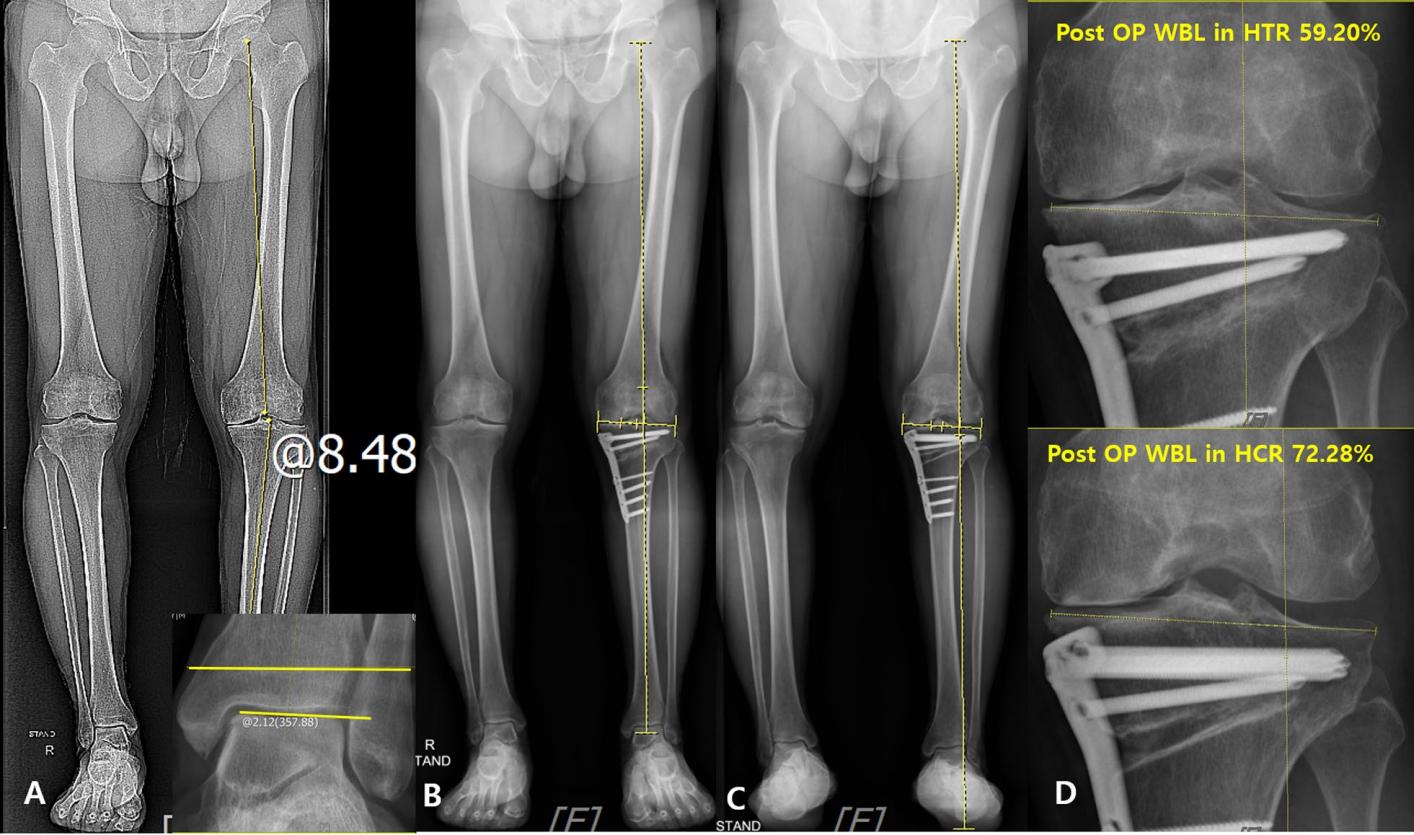
**A** 66-year-old male patient showed left knee osteoarthritis with hip-knee-ankle angle 8.4° genu varum deformity. The ankle joint line obliquity was 2.12°. **B** The conventional mechanical axis after opening-wedge high tibial osteotomy (OWHTO). **C** The true mechanical axis after OWHTO. **D**. The postoperative weight-bearing line (WBL) ratio in the hip-to-talus radiograph after OWHTO was 59.2%, and that of the WBL ratio in the hip-to-calcaneus radiograph after OWHTO was 72.2%. The true mechanical axis showed more lateral position compared to the conventional mechanical axis after OWHTO

Our results showed that preoperative AJLO and LDTGA were significantly lower in the inconsistent group than those in the consistent group. Preoperative AJLO was a significant factor affecting the inconsistency in the WBL ratio and was negatively correlated with it. Therefore, we believed that a smaller preoperative AJLO can result in a higher possibility of inconsistency of the WBL ratio. Varus malalignment of the knee joint leads to valgus compensation of the ankle and subtalar joints [11–13]. The compensation of the subtalar joint has been reported more frequently than that of the ankle joint [12]. However, if the compensation in the subtalar joint is inappropriate, the talus inclination would not change properly in response to the tibial plafond inclination, resulting in less preoperative AJLO. This explanation applies to our finding that the preoperative AJLO was a significant risk factor for the inconsistency in the WBL ratio and was negatively correlated with the inconsistency between the two radiographs. Therefore, we believe that evaluating preoperative AJLO would be helpful in predicting the possibility of a more valgus alignment in the true mechanical axis after OWHTO (Figs. 6, 7). In such cases, it is important to be cautious not to make overcorrect > 5% of the WBL ratio even if the proper alignment is achieved in the HTR after OWHTO.

Our results also found that the inconsistent group had a significantly smaller postoperative AJLO than the consistent group. The postoperative AJLO can be used to evaluate the compensation status and stiffness of the subtalar joint [39]. Yamasaki et al. divided patients who underwent TKA into two groups based on postoperative AJLO (postoperative AJLO < ± 1° and postoperative AJLO > ± 1°). The group with a postoperative AJLO < ± 1° was considered to have good compensation ability [39]. However, this was inconsistent with our results. TKA requires a neutral alignment and strict medial and lateral balancing of the knee joints. If the compensation occurs properly, the postoperative AJLO can be in a nearly horizontal position [39]. However, when performing OWHTO, we need to consider the possibility of a change in the knee joint according to the status of the ligament of the knee joint, which would cause a difference in the status of postoperative AJLO between TKA and HTO.

This study had several limitations. First, it was a retrospective design and only analyzed the short-term follow-up outcomes. Only a small number of patients were included in this study. However, the number of patients exceeded the minimum number of patients required for the post hoc analysis to show a power of 0.8. Second, we did not assess hindfoot alignment directly, although we could estimate the flexibility of the subtalar joint through this evaluation. Third, although the knee deformity was three-dimensional, we only used two-dimensional images. In addition, there may have been errors in taking the radiographs; however, we standardized the process to reduce the occurrence of errors, as described in our previous study [10]. Fourth, in previous studies, the true and conventional mechanical axes were evaluated only for the HCR. However, in our study, we evaluated the conventional mechanical axis in HTR and the true mechanical axis in HCR. Because we usually performed preoperative planning and postoperative evaluation of the HTR, we believe that it is appropriate to evaluate each mechanical axis on different radiographs. However, we do not believe that the mechanical axis of HCR could entirely supplant the mechanical axis of HTR and the measurement around the knee joint. What we aim to emphasize through this study is the necessity of taking into account the actual weight-bearing point when evaluating lower limb alignment additionally. This becomes particularly critical in patients with a small AJLO before undergoing OWHTO surgery, necessitating careful consideration of this aspect.

## Conclusion

The pre-and postoperative AJLO and preoperative LDTGA were significantly different between the consistent and inconsistent groups. Among these variables, only the preoperative AJLO negatively affected the inconsistency of WBL ratios between the two types of radiographs (HTT and HTC). However, short-term clinical outcomes did not differ significantly between the two groups. Therefore, it should be considered to prevent postoperative overcorrection of the true mechanical axis after OWHTO, even though we corrected it properly. Further studies are warranted to analyze whether the inconsistency between the two radiographs would affect the longer-term clinical outcomes.

### Supplementary Information

**Additional file 1. Appendix**. Reliability analysis of radiographic variables.

## Data Availability

Not applicable.
