# Supplementary material for: Analysis of radiographic factors affecting the significant differences in knee alignment between hip-to-talus and hip-to-calcaneus radiographs after opening-wedge high tibial osteotomy
Source: Knee Surg Relat Res. 2023 Dec 7;35:28. doi: 10.1186/s43019-023-00203-4 (PMC10704678; doi:10.1186/s43019-023-00203-4)
Supplement: Supplementary file 1 — Additional file 1. Appendix. Reliability analysis of radiographic variables. [file 43019_2023_203_MOESM1_ESM.docx]

Appendix. Reliability analysis of radiographic variables.

|  | Inter-observer correlation coefficient | Intra-observer correlation coefficient | |
| --- | --- | --- | --- |
| **Preoperative** |  |  |  |
| Lower extremity alignment | | |  |
| HKA | 0.995 | 0.996 |  |
| mLDFA | 0.979 | 0.976 |  |
| MPTA | 0.961 | 0.975 |  |
| mLDFA | 0.836 | 0.847 |  |
| Knee |  |  |  |
| JLCA | 0.994 | 0.986 |  |
| KJLO | 0.992 | 0.987 |  |
| PTS | 0.975 | 0.971 |  |
| Ankle |  |  |  |
| TTA | 0.992 | 0.972 |  |
| AJLO | 0.998 | 0.995 |  |
| LDTGA | 0.998 | 0.996 |  |
|  |  |  |  |
| **Postoperative** |  |  |  |
| Lower extremity alignment | | |  |
| HKA | 0.999 | 0.992 |  |
| mLDFA | 0.997 | 0.967 |  |
| MPTA | 0.998 | 0.984 |  |
| mLDFA | 0.993 | 0.976 |  |
| Knee |  |  |  |
| JLCA | 0.999 | 0.982 |  |
| KJLO | 1.000 | 0.993 |  |
| PTS | 1.000 | 0.971 |  |
| Ankle |  |  |  |
| TTA | 0.994 | 0.984 |  |
| AJLO | 0.998 | 0.966 |  |
| LDTGA | 0.997 | 0.970 |  |
|  |  |  |  |
| **Difference *** |  |  |  |
| Lower extremity alignment | | |  |
| HKA | 0.995 | 0.991 |  |
| mLDFA | 0.896 | 0.866 |  |
| MPTA | 0.974 | 0.974 |  |
| mLDFA | 0.961 | 0.939 |  |
| Knee |  |  |  |
| JLCA | 0.984 | 0.920 |  |
| KJLO | 0.990 | 0.965 |  |
| PTS | 0.969 | 0.930 |  |
| Ankle |  |  |  |
| TTA | 0.892 | 0.802 |  |
| AJLO | 0.991 | 0.936 |  |
| LDTGA | 0.994 | 0.955 |  |

Values are presented as mean ± standard deviation.

* Difference between postoperative and preoperative values

Abbreviation: HKA, hip knee ankle axis; MPTA, medial proximal tibial angle, JLCA, joint line convergence angle; KJLO, knee joint line obliquity; PTS, posterior tibial slope, TTA, talar tilt angle; AJLO, ankle joint line obliquity; LDTGA, lateral distal tibial ground surface angle

Statistical analysis: reliability analysis
